# Supplementary material for: Creating a ‘Molecular Band-Aid’; Blocking an Exposed Protease Target Site in Desmoplakin
Source: J Pers Med. 2021 May 12;11(5):401. doi: 10.3390/jpm11050401 (PMC8151963; doi:10.3390/jpm11050401)
Supplement: Supplementary file 1 [file jpm-11-00401-s001.zip › jpm-1175153-supplementary-final.pdf]

Table S1. List of intramolecular interactions stabilizing the SH3/SR4 interface, important in occluding the calpain target site in proximal variants in the presence of L518Y.

**Residue and Atom Interaction**
Asp 300 Atom CG—Lys 449 Atom NZ
Pro 450 Atom O—Asn 487 Atom ND2
Asp 300 Atom CG—Asn 452 Atom ND2
Leu 448 Atom O—Asp 300 Atom H
Ser 507 Atom HG—Arg 490 Atom O
Ser 507 Atom O—Arg 451 Atom CZ
Ser 507 Atom OG—Arg 451 Atom CZ
Leu 510 Atom O—Arg 451 Atom CZ
Asn 487 Atom ND2—Asn 452 Atom O
Asp 297 Atom CG—Ser 299 Atom HG
Ser 299 Atom O—Asn 302 Atom H
Lys 301 Atom H—Ser 299 Atom OG
Lys 449 Atom O—Asn 452 Atom H
Leu 448 Atom O—Arg 451 Atom H

Table S2. List of additional calpain interactions between residues 448–449, in the middle of the calpain target site, and residues on the SR4 loop (residues 299–300).

Residue and Atom Interaction
Gln 447 Atom NE2—Pro 515 Atom O
Gln 447 Atom NE2—Pro 513 Atom O
Lys 449 Atom NZ—Asp 300 Atom CG
Arg 451 Atom CZ—Ser 507 Atom O
Arg 451 Atom CZ—Ser 507 Atom OG
Asn 452 Atom ND2—Ser 299 Atom OG
Asn 452 Atom OD1—Lys 301 Atom NZ
Asn 452 Atom ND2—Asn 300 Atom CG

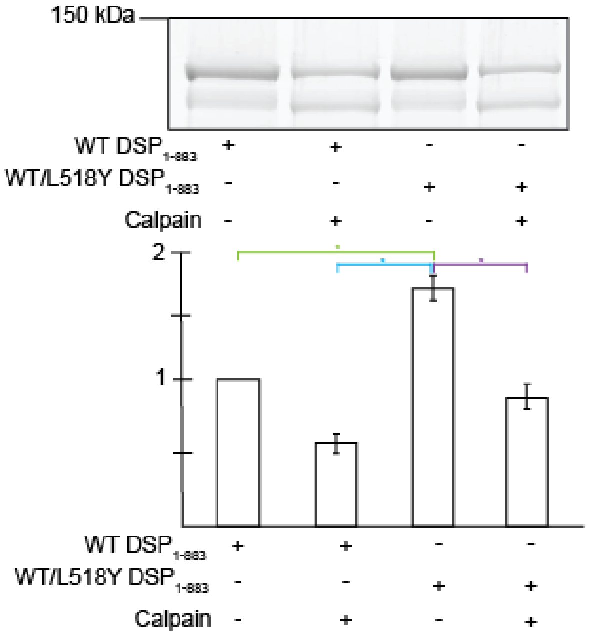

Figure S1. The effect of the L518Y mutation on WT DSP. Gel is representative. Graphs show protein recovery ratios as determined by densitometry. Data are represented by the mean +/- SEM. Statistics were performed with 1-way ANOVA and the Tukey HSD test was used to determine significance. Colored bars represent significance; $p < 0.05$. WT Set, $n = 5$.

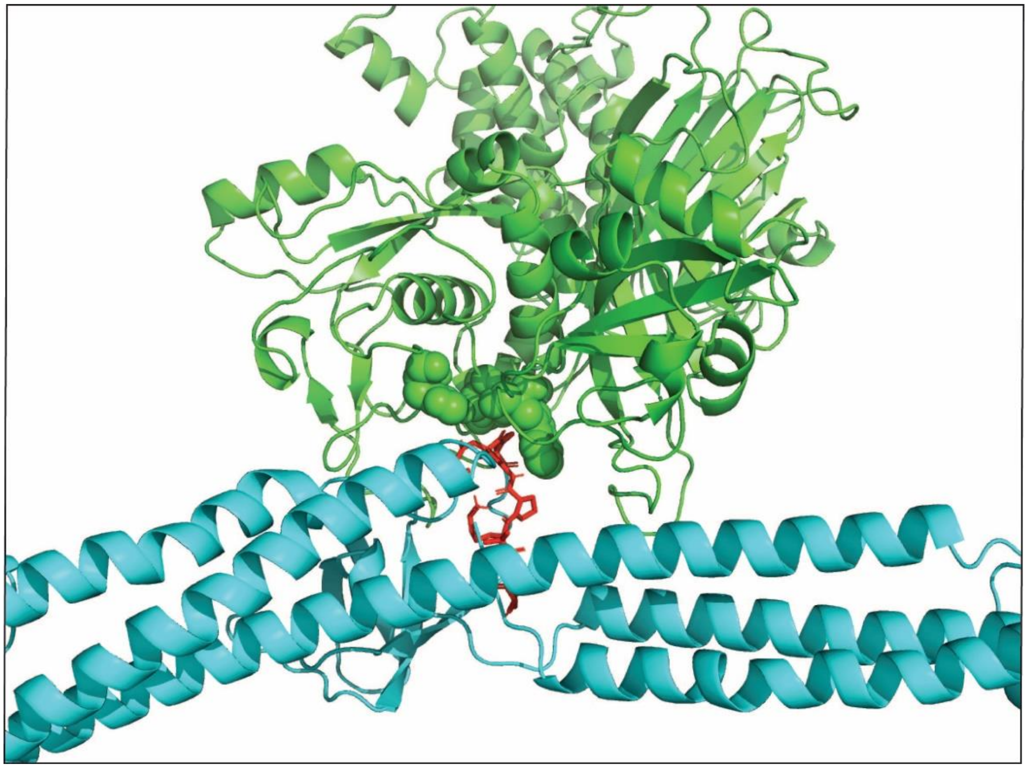

Figure S2. Model of calpain bound to desmoplakin: m-calpain (in green; derived from pdb 3DF0) [40] contains a shallow binding cleft (spheres) that DSP 447–453 (in red) can be docked into using the program HADDOCK [41]. DSP 447-453 must partially unfold to successfully dock to m-calpain. This dynamic local event of unfolding is strongly suggested from the increased solvent accessibility and decreased interactions of the DSP mutants studied here. Furthermore, the presence of L518Y guards against the likelihood that such an unfolding event will occur.

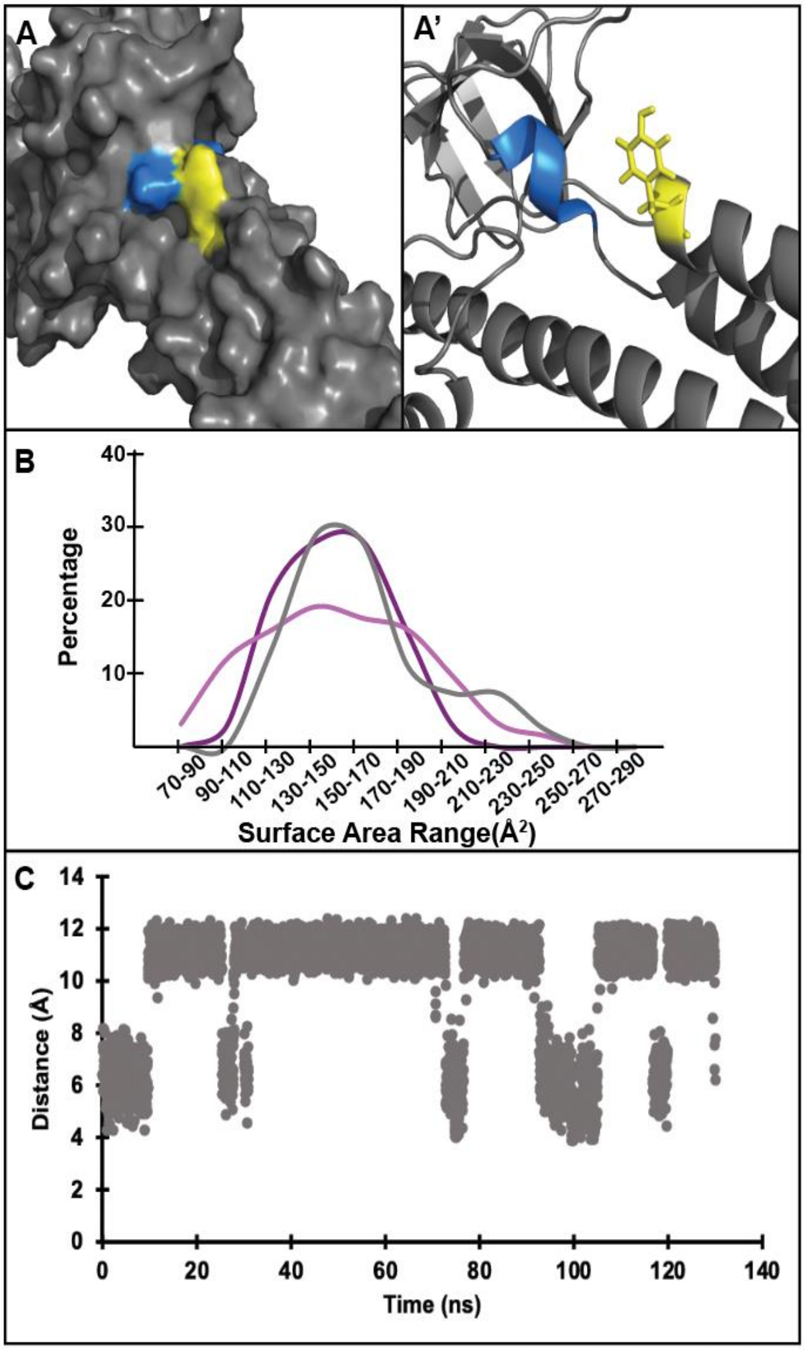

Figure S3. L518Y partially blocks the exposed DSP target cleavage site in the presence of S299R. (**A**,**A'**) L518Y covers part of the calpain target site in the 'flipped in' position. (**B**) MD simulations of S299R/L518Y show that inclusion of L518Y decreases the solvent exposure of the calpain target site. (**C**) L518Y in S299R spends roughly 22% of the time in the 'flipped in' position, as determined by the distance of L518Y from P517.

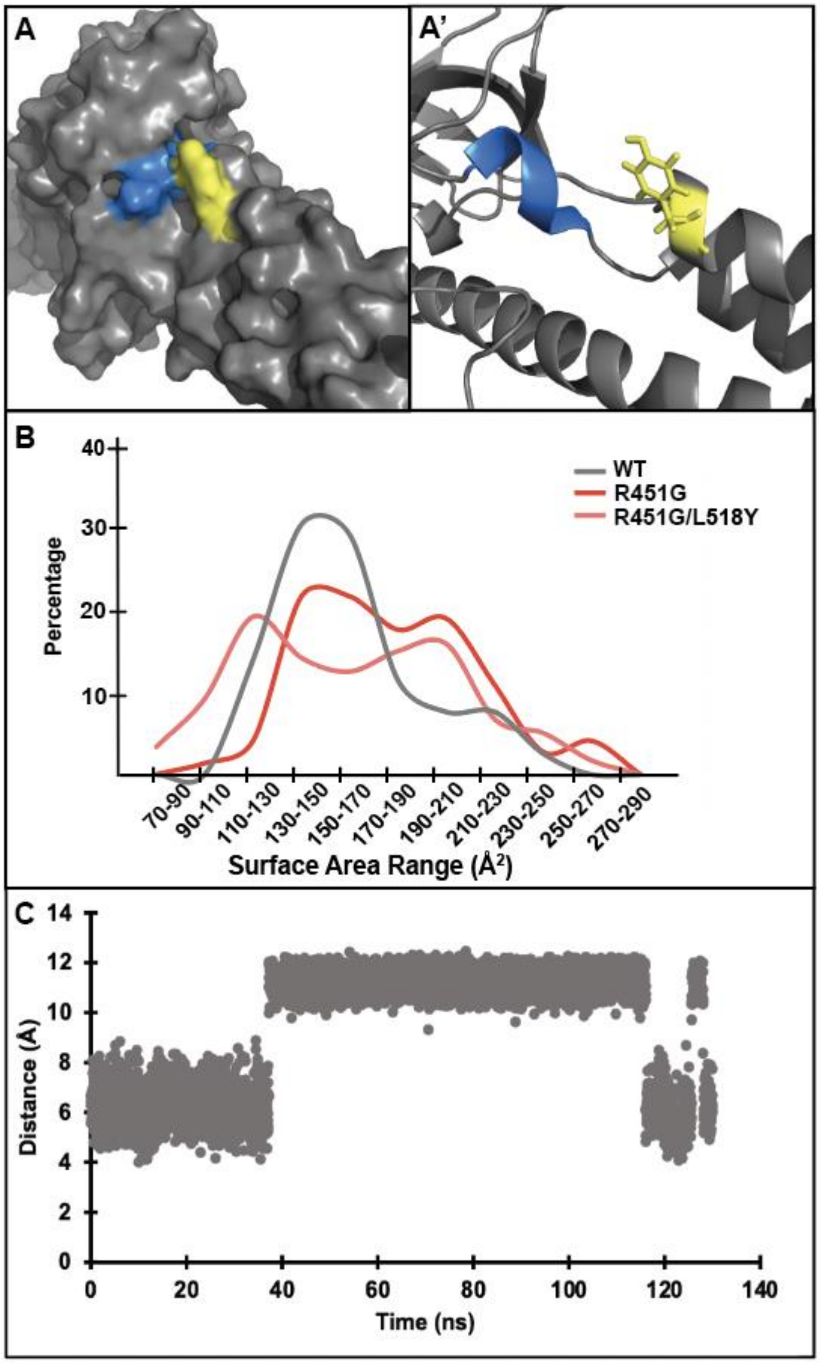

Figure S4. L518Y partially blocks the exposed DSP calpain target site in the presence of R451G. (**A**,**A'**) L518Y covers part of the calpain target site in the 'flipped in' position. (**B**) MD simulations of R451G/L518Y show that inclusion of L518Y decreases the solvent exposure of the calpain target site. (**C**) L518Y in R451G spends roughly 38% of the time in the 'flipped in' position, as determined by the distance of L518Y from P517.

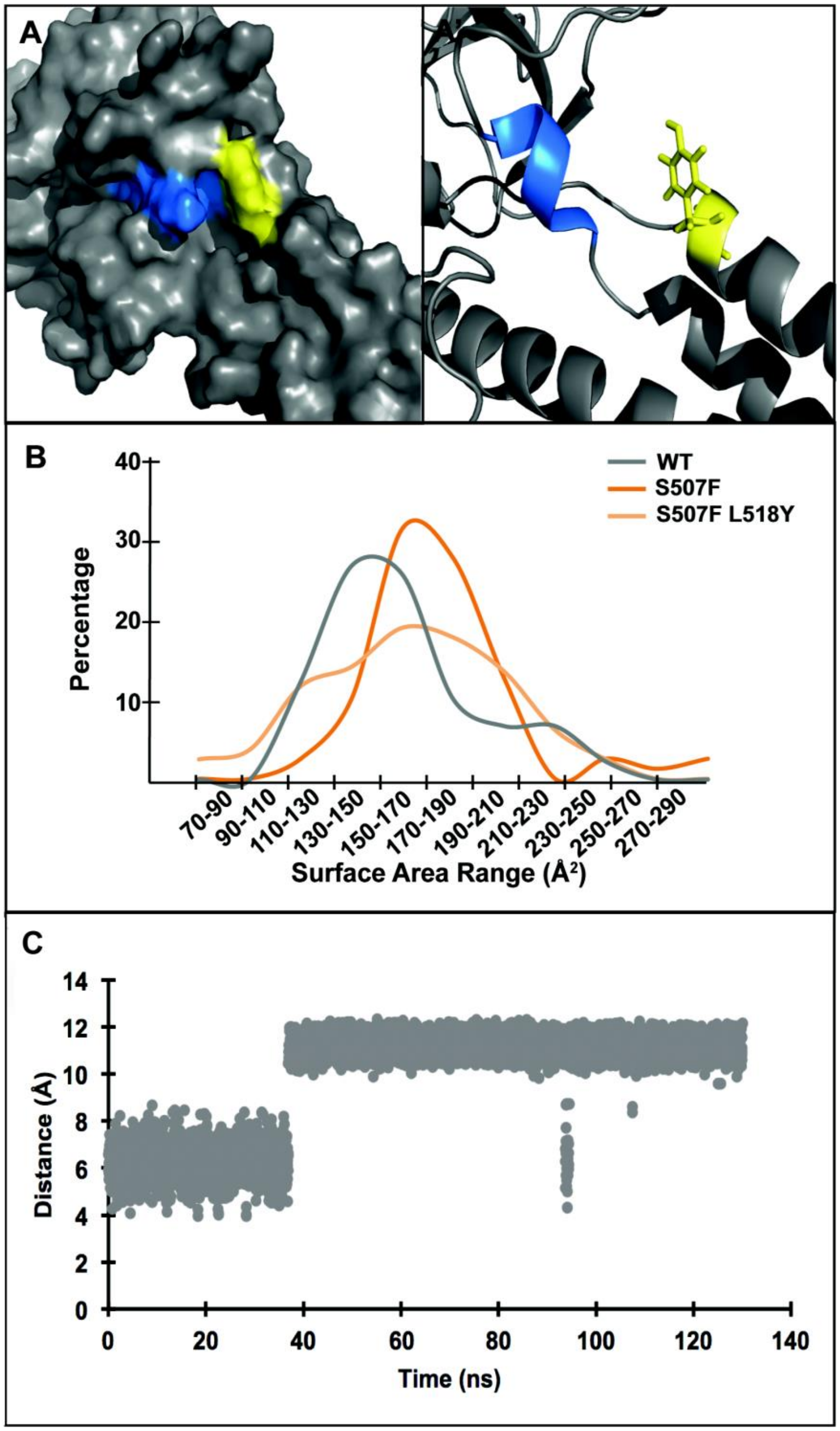

Figure S5. L518Y partially blocks the exposed DSP calpain target site in the presence of S507F. (**A**,**A'**) L518Y covers part of the calpain target site in the 'flipped in' position. (**B**) MD simulations of S507F/L518Y show that inclusion of L518Y decreases the solvent exposure of the calpain target site. (**C**) L518Y in S507F spends roughly 26% of the time in the 'flipped in' position, as determined by the distance of L518Y from P517.
